# The brachial plexus branches to the pectoral muscles in adult rats: morphological aspects and morphometric normative data

**DOI:** 10.3389/fnana.2012.00041

**Published:** 2012-10-10

**Authors:** Nilo Riva, Teuta Domi, Ignazio Diego Lopez, Daniela Triolo, Andrea Fossaghi, Giorgia Dina, Paola Podini, Giancarlo Comi, Angelo Quattrini

**Affiliations:** ^1^Neuropathology Unit, Division of Neuroscience and INSPE, San Raffaele Scientific InstituteMilan, Italy; ^2^“Vita e Salute” San Raffaele UniversityMilan, Italy

**Keywords:** motor fibers, peripheral nerve, brachial plexus, pectoral nerves, medial anterior thoracic nerve, lateral anterior thoracic nerve, rat anatomy

## Abstract

Animal models provide an important tool to investigate the pathogenesis of neuromuscular disorders. In the present study, we analyze fiber composition of the brachial plexus branches to the pectoral muscles: the medial anterior thoracic nerve (MATN) and the lateral anterior thoracic nerve (LATN). The morphological and morphometric characteristics and the percentage of motor fibers within each nerve are here reported, adding information to microscopic anatomy knowledge of the rat brachial plexus. As control, we employed the quadriceps nerve, commonly used for the evaluation of motor fibers at hindlimbs. We demonstrated that the MATN and the LATN are predominantly composed of large motor fibers and therefore could be employed to evaluate the peripheral nervous system (PNS) involvement at forelimbs in neurological diseases models, predominantly affecting the motor fiber compartment.

## Introduction

Each peripheral nerve is composed of a different percentage of motor, sensory, and autonomic fibers. Therefore, the knowledge of the morphological features of peripheral nerves is of primary importance (Schaumburg et al., 2010) for the comprehension of the pathogenesis of diseases affecting the peripheral nervous system (PNS) (De Medinacelli, 1995).

Although PNS diseases can affect all fibers, motor fibers are selectively or predominantly involved in different neuromuscolar disorders, including neuron diseases (MND), spinal muscular atrophies (SMA), and different types of motor neuropathies (Riva et al., 2011).

In animal models, quadriceps nerve is commonly used in order to assess the motor fiber involvement at hind-limbs (Kobsar et al., 2003; Grohmann et al., 2004). In contrast, at forelimbs a predominantly motor nerve has yet to be indentified and systematically characterized. In rat anatomy, the brachial plexus branches supplying the pectoral muscles are the medial anterior thoracic nerve (MATN), which originates from the eighth cervical and the first thoracic nerves, and the lateral anterior thoracic nerve (LATN), which originates from the sixth and seventh cervical nerves. The MATN also innervates the cutaneous maximus muscle, which is not conserved in human anatomy (Greene, 1955; Matsuda et al., 1995). Here we characterized the fiber composition of the MATN and the LATN in Sprague-Dawley adult rats, showing that these nerves are composed mainly of large motor fibers. Thus, MATN and LATN histopathological analysis could be useful to asses motor fiber involvement in animal models.

## Materials and methods

### Animals

Studies were conducted in 20 adult (4-month-old) female Sprague-Dawley rats (Charles River Lab). All experiments were carried out following Italian regulations and in accordance with the S. Raffaele Institutional Animal Care and Use Committee.

### Preparation and histological processing of samples

**Figure 1:**
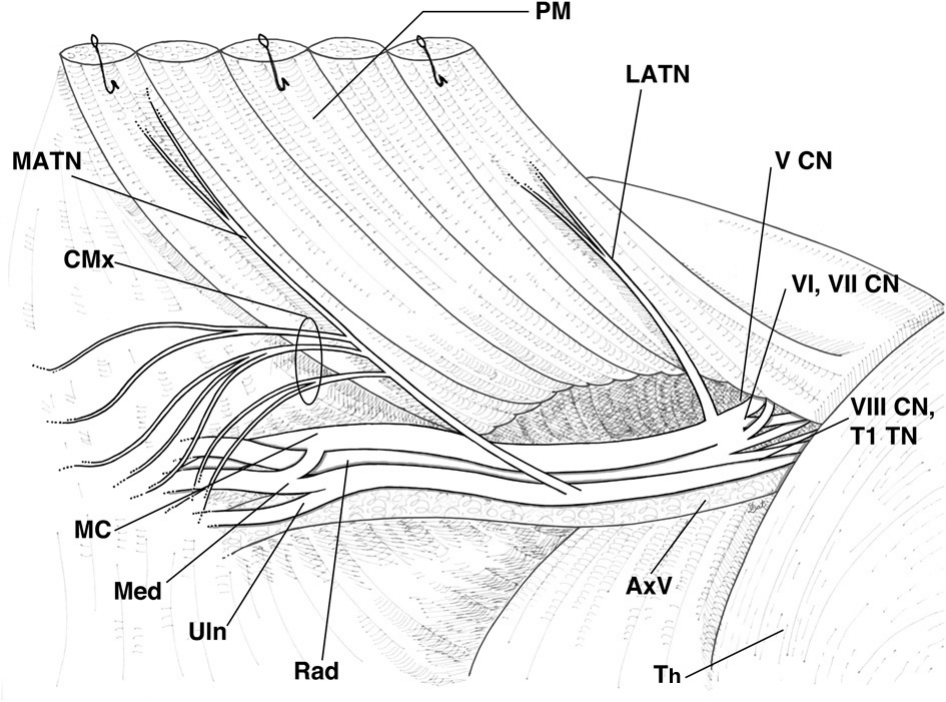
**Schematic representation of the right rat brachial plexus highlighting the relations of the lateral and medial anterior thoracic nerves**. PM, pectoral muscles, reflected; MATN, medial anterior thoracic nerve; CMx, branches of the MATN to the cutaneous maximus muscle; LATN, lateral anterior thoracic nerve; V CN, fifth cervical nerve; VI, VII CN, sixth and seventh cervical nerves; VIII CN, TI TN, eighth cervical nerve and first thoracic nerve; Th, thorax; AxV, axillary vessels; Rad, radial nerve; Uln, ulnar nerve; Med, median nerve; MC, muscolocutaneous nerve.

Animals were sacrificed by CO_2_ inhalation. In 10 rats, the MATN and LATN were harvested: the skin and fascia were removed throughout the thoracic and forelimb areas; the pectoralis major muscle was detached from the sternum and the first ribs, while the pectoralis minor muscle was detached from the rib cage; muscle bellies were then carefully reflected superolaterally and the brachial plexus exposed. MATN and LATN were then dissected distally toward their terminations. This approach allowed all muscular attachments to be maintained. The quadriceps nerve, obtained from 10 rats, was employed as control (Figure 1).

Each nerve was harvested bilaterally; the MATN was harvested distal to the branches to the cutaneous maximus muscle. One nerve was fixed in 2% buffered gluteraldehyde and post-fixed in 1% osmium tetroxid. After alcohol dehydration, samples were embedded in Epon; transverse sections (0.5–1 mm) were stained with toluidine blue and examined by light microscopy. The contra-lateral nerve was fixed by immersion in 4% paraformaldehyde in 0.1 M phosphate-buffered saline (PBS), cryoprotected with sucrose, embedded in OCT (Leica Microsystems Nussloch GmbH), and snap-frozen in liquid nitrogen (Quattrini et al., 1996; Wrabetz et al., 2000).

### Morphometric analysis

Digitalized, images of fiber cross-sections were obtained from corresponding levels of the MATN, LATN, and quadriceps nerves with a digital camera (Leica DFC300F, Milano, Italy) using a 100× objective. For each nerve, at least five non-overlapping images from 10 different animals were acquired (30 × 10^3^ μm^2^ of nerve per each animal). Myelinated fibers morphometry on semi-thin sections was analyzed with the Leica QWin software (Leica Mycrosystems, Milano, Italy) (Triolo et al., 2006). Only fibers of circular shape were measured. The g-ratio, a measure of the degree of myelination, defined as the ratio between the mean diameter of an axon and the mean diameter of the fiber, including myelin, was determined on at least 150 randomly chosen fibers per nerve (10 samples per each different nerve). Distribution spectra of myelinated fibers, separated into 1 μm diameter class intervals, were constructed from both axonal and total fiber diameters (Scott, 1979). The total number of fibers and the transverse sectional areas of the fascicles were quantified using ImageJ software (US National Institutes of Health).

### Immunohistochemical analysis

Immunostaining was performed on 10-μm thick cryosections that were sequentially incubated in: peroxidase blocking solution (3% H_2_O_2_) for 5 min at room temperature (RT); TBS containing 0.05% Tween for 5 × 2 min; purified anti-goat anti-choline acetyltransferase (ChAT) antibody, a marker for motor fibers (Lago and Navarro, 2006), (1:100, Millipore AB144P) for 60 min at RT; TBS containing 0.05% Tween for 5 × 2 min; goat-on-rodent HRP polymer (Biocare Medical GP626H) for 60 min at RT; TBS containing 0.05% Tween for 5 × 2 min and working solution of the metal-enhanced diaminobenzidine (DAB) substrate kit (Thermo Scientific). After rinses in distilled water, sections were dehydrated through scale of ethanol (70, 95, 100%, 1 min each), cleared in xylene for 20 min, mounted on untreated clean glass slides and covered with mounting medium (Micromount, Bioptica) and a glass cover slip. Sections were examined by light microscope (Olympus BX51, Segrate, Italy). Digitalized images of fiber cross-sections were obtained from corresponding levels of each nerve with a digital camera (Leica DFC300F, Milano, Italy) using a 40× objective. ChAT positive and negative fibers were counted for each nerve, and the percentage of ChAT positive fibers calculated. Two adult rat ventral and dorsal roots were used as positive and negative controls, respectively.

### Statistical analysis

All results are presented as mean ± standard deviation (SD). Differences between nerves of the mean values of morphometric parameters and the percentage of ChAT-positive nerve fibers have been analyzed by One-Way ANOVA followed by Bonferroni *post-hoc* test. Differences in fiber diameter distribution were further analyzed using repeated measures ANOVA with NERVE (quadriceps, MATN, and LATN) as the between subject factor and FIBER DIAMETER (the percentage of nerve fibers for each 1 μm diameter class intervals, from 1 to 16 μm) as the within subject factor. When the sphericity assumption, verified using Mauchly's criterion, was rejected, the Greenhouse–Geisser correction was performed. *Post-hoc* analysis was carried out with the Bonferroni test, in order to detect significant differences in nerve fiber diameter composition between nerves. Statistical significance was considered at *p* < 0.05. All statistical tests were performed using SPSS software (Technologies, Inc., Chicago, IL, USA).

## Results

### Morphological findings

**Figure 2:**
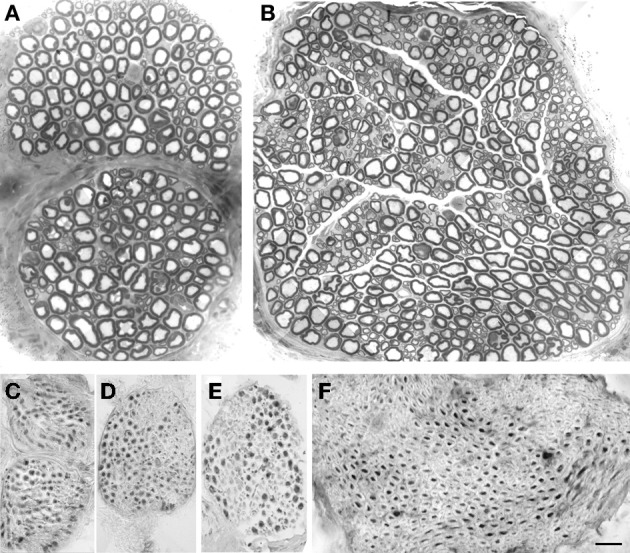
**Morphological and immunohistochemical studies of the branches of the brachial plexus to the pectoral nerves**. Semithin sections showing fiber distribution in MATN **(A)** and LATN **(B)**. Large fibers are prevalent in MATN **(A)** compared with LATN **(B)**, in which fiber distribution is bimodal. ChAT immunohistochemical analysis showed that almost all fibers are positive in MATN **(C** and **D)**, compared to LATN **(E)** and quadriceps **(F)**. BAR: **(A** and **B)**: 15 μm; **(C–F)**: 30 μm. Adobe Photoshop v. 8 was used to size, and crop micrographs, and to construct plates. Images were enhanced only for contrast and brightness.

In all animals, the MATN and LATN were properly identified and isolated (Figure 1). Macroscopically, about 3–5 mm distal to the branches to the cutaneous maximus muscle, the MATN invariably divided into two branches, coursing along the undersurface of the pectoralis muscles. The LATN was in all cases constituted by a single nerve branch, about 8–10 mm long, distally dividing into 3–6 intramuscular branches. Semithin sections inspective analysis revealed that the MATN was largely composed of large diameter fibers (Figure 2A), while in the LATN a non-negligible amount of small and medium size diameter fibers was also present (Figure 2B). This fiber population could also be detected, to a lesser extent, in the quadriceps, our control nerve.

### Myelinated fiber morphometry

The results of morphometric analysis of myelinated fibers are shown in Table 1. The MATN proved the smallest nerve. One-Way ANOVA yielded a significant difference between nerves with regard both to fiber number [*F*_(2, 27)_ = 51.72, *p* < 0.0001], and endoneurial area [*F*_(2, 27)_ = 34.68, *p* < 0.0001]. *Post-hoc* comparison showed that mean fiber number and endoneurial area were significantly lower in the MATN compared with both the LATN and quadriceps nerves (*p* < 0.0001) and in the LATN compared with the quadriceps nerve (*p* < 0.05).

**Table 1:** **Morphometric parameters of myelinated fibers of the lateral and medial anterior thoracic and quadriceps nerves**.

	**Quadriceps**	**Medial anterior thoracic**	**Lateral anterior thoracic**
Number of fibers	1075.0 ± 142.4	410.9 ± 113.4^***^^;^^###^	872.5 ± 184.6^*^
Endoneurial area (μm^2^)	122,737 ± 21,917	45,441 ± 16,690^***^^;^^#^	74,835 ± 23,618^***^
Myelinated fiber density (fibers/mm^2^)	9247 ± 1417	9240 ± 1407	11,976 ± 3996
Mean fiber diameter (μm)	8.52 ± 3.36	9.26 ± 3.34^***^^;^^###^	7.76 ± 3.59^***^
Mean axon diameter (μm)	5.95 ± 2.64	6.24 ± 2.49^**^^;^^###^	5.29 ± 2.63^***^
g-Ratio	0.700 ± 0.066	0.703 ± 0.063	0.700 ± 0.067
% ChAT+	67.0% ± 10.1%	88.8% ± 5.1%^***^^;^^###^	61.2% ± 7.7%

Data are mean ± SD. % ChAT+, percentage of choline acetyltransferase-positive fibers.

* p < 0.05 vs. quadriceps;

** p < 0.005 vs. quadriceps;

*** p < 0.0005 vs. quadriceps.

# p < 0.05 vs. lateral anterior thoracic;

### p < 0.0005 vs. lateral anterior thoracic.

Morphometrical analysis confirmed that the MATN was composed of a population of fibers of larger dimensions compared with the other nerves, while the lowest values were recorded for the LATN (Table 1). One-Way ANOVA showed a significant difference between nerves in regard to both fiber and axon mean diameters [*F*_(2, 6772)_ = 111.78, *p* < 0.0001 and *F*_(2, 6772)_ = 85.51, *p* < 0.0001, respectively]. *Post-hoc* comparison showed significantly higher mean fiber and axon diameter for the MATN compared with both the LATN (*p* < 0.0001), and quadriceps nerves (*p* < 0.005); while they were significantly lower in the LATN compared with the quadriceps nerve (*p* < 0.0001). The g-ratio was not different between nerves (Table 1). No significant fiber density differences were found.

**Figure 3:**
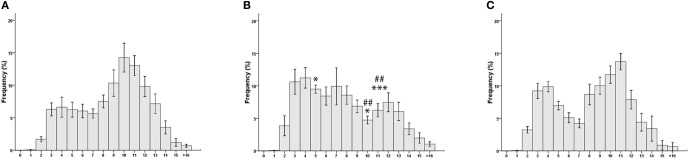
**Morphometrical analysis results**. Myelinated fibers distribution spectra: **(A)** medial anterior thoracic nerve; **(B)** lateral anterior thoracic nerve; **(C)** Quadriceps nerve. ^*^p < 0.05 vs. quadriceps; ^***^p < 0.005 vs. quadriceps. ^##^*p* < 0.005 vs. lateral anterior thoracic.

Myelinated fibers distribution spectra are shown in Figure 3. In the MATN the population of low-diameter fibers, including sensory fibers, is reduced; as a consequence, the myelinated fiber distribution histogram is shifted to the right (Figure 3A). In the LATN (Figure 3B), large-diameter fibers, largely composed of motor fibers, are less represented compared with the other nerves. In the quadriceps nerves, our control nerve, a bimodal distribution of fibers is observed (Figure 3C).

The percentages of each single fiber-diameter class are shown in Table 2. Repeated measures ANOVA, carried out in order to detect differences in the percentage of single fiber-diameter classes, yielded a significant interaction between the factors NERVE and FIBER DIAMETER [*F*_(30, 405)_ = 3067, *p* = 0.003]. *Post-hoc* analysis showed that the percentage of fibers of 5–6 μm of diameter was significantly higher in the LATN compared with the MATN (9.5 and 6.2%; *p* = 0.033), but not with the quadriceps nerve (6.9%). On the contrary, the percentage of fibers of 10–11 μm and 11–12 μm of diameter was significantly lower in the LATN (4.7 and 6.2%, respectively) compared with both the MATN (14.2 and 13.0%; *p* < 0.0001 and *p* = 0.003, respectively) and the quadriceps nerves (11.7 and 13.7%; *p* < 0.010 and *p* = 0.001, respectively).

**Table 2:** **Distribution spectra of myelinated fibers of pectoral and quadriceps nerves**.

	**1–2 (%)**	**2–3 (%)**	**3–4 (%)**	**4–5 (%)**	**5–6 (%)**	**6–7 (%)**	**7–8 (%)**	**8–9 (%)**	**9–10 (%)**	**10–11 (%)**	**11–12 (%)**	**12–13 (%)**	**13–14 (%)**	**14–15 (%)**	**15–16 (%)**	**>16 (%)**
Medial anterior thoracic	0.1	1.7	6.3	6.6	6.2^#^	6.0	5.6	7.5	10.3	14.2^##^	13.0^##^	9.8	7.1	3.5	1.2	0.7
Lateral anterior thoracic	0.0	3.9	10.6	11.2	9.5	8.4	9.9	8.6	6.9	4.7^*^	6.2^***^	7.5	6.1	3.4	2.0	1.1
Quadriceps	0.1	1.7	6.3	6.6	6.9	6.0	5.6	7.5	10.3	11.7	13.7	9.8	7.1	3.5	1.2	0.7

Data are mean percentage of fibers, for each 1 μm diameter class interval.

* p < 0.05 vs. quadriceps;

*** p < 0.0005 vs. quadriceps.

# p < 0.05 vs. lateral anterior thoracic;

## p < 0.005 vs. lateral anterior thoracic.

### Immunohistochemistry

Immunohistochemical studies of transverse sections of the MATN, LATN, and quadriceps nerves demonstrated that in these nerves most fibers are ChAT positive (Figures 2C–F). Inspective analysis revealed that motor, ChAT-positive fibres were mainly represented by intermediate and large diameter fibers, while most sensory, ChAT negative fibers were of low and intermediate diameter. ChAT-positive and ChAT-negative fibers were grouped together within the fascicles, thus displaying a non-uniform distribution within the endonerium.

**Figure 4:**
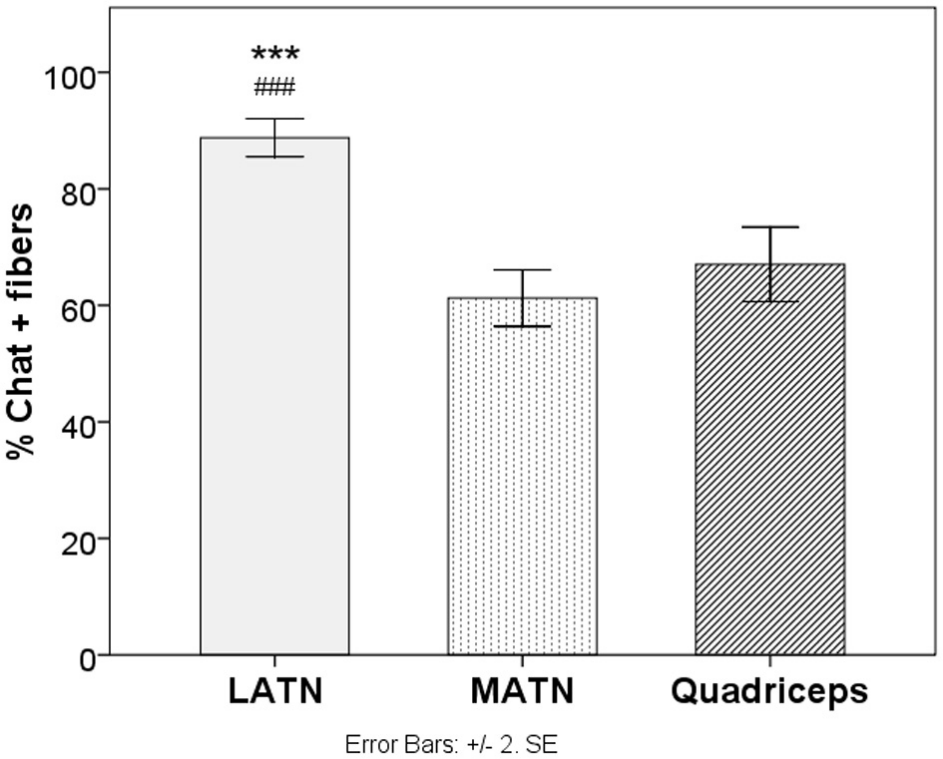
**Immunohistochemical analysis**. Quantitative analysis of the percentage of ChAT positive fibers in study nerves. LATN, Lateral Anterior Thoracic Nerve; MATN, medial anterior thoracic nerve. ^***^p < 0.005 vs. quadriceps. ^###^*p* < 0.005 vs. lateral anterior thoracic.

The mean percentage of ChAT-positive fibers was 88.8% for the MATN, 61.2% for the LATN, and 67.0% for the quadriceps nerve (Table 1). One-Way ANOVA yielded a significant difference between nerves in the percentage of ChAT-positive fibers [*F*_(2, 27)_ = 51.72, *p* < 0.0001]. *Post-hoc* comparison showed that the percentage of ChAT-positive fibers was significantly higher in the MATN compared with both the LATN and quadriceps nerves (*p* < 0001), while the difference between the LATN and quadriceps nerve did not reach statistical significance (Figure 4).

## Discussion

We report for the first time a systematic description, including morphometrical and immunohistochemical analysis, of the branches to the pectoral muscles of the rat brachial plexus, showing that these nerves are predominantly composed of large motor nerve fibers.

Isolation of the MATN and LATN proved a simple and reproducible procedure and the nerves could be easily identified and harvested in all study animals. Since in the rat, contrary to humans, the entire brachial plexus is located infraclavicularly (Bertelli and Ghizoni, 2006), our surgical approach allowed the exposure of the entire plexus with maintenance of all muscular attachments of the MANT and LATN.

The MATN, harvested distal to the branches to the cutaneous maximus nerve, proved the smallest of the nerves studied regarding both endoneurial area and number of fibers. While no terms of comparison are available for the MATN and the LATN, our data concerning quadriceps nerve fiber numbers are in line with previous literature reports (Peyronnard et al., 1986). Mean fiber and axonal diameters were significantly higher in the MATN compared with both LATN and quadriceps nerves, while they were significantly higher in the quadriceps nerve compared with the LATN. The LATN is considered to correspond in human anatomy to the lateral pectoral nerve, which originates from the lateral cord of the brachial plexus, while the MATN corresponds to the medial pectoral nerve, from the medial cord of the brachial plexus. In a human autopsy study, however, it has been shown that the pectoral nerves would exist at the trunk level as 3 distinct nerves: the superior, middle, and inferior pectoral nerves; the mean fiber diameter was similarly high in the middle and inferior pectoral nerves (8.83 and 8.53 μm, respectively), while it was lower in the superior pectoral nerve (7.21 μm) (Aszmann et al., 2000). In our study, performed on the rat brachial plexus, we could not identify the existence of three pectoral nerves, thus confirming previous reports (Greene, 1955), and suggesting that in the rat the middle pectoral nerve of human anatomy could be absent or fused to the LATN or the MATN. In both species, however, these nerves are composed of large-diameter fibers.

Myelinated fibers distribution spectra visual analysis confirmed a shift to the right for the MATN and the quadriceps nerve, similarly to ventral motor roots (Dyck et al., 1982). Moreover, direct comparison of the percentage of single fiber-diameter classes confirmed a higher percentage in the MATN and quadriceps nerve of large diameter fibers, namely of 10–11 μm and 11–12 μm of diameter. As large diameter fibers mainly derive from alpha motor neurons, the above-mentioned findings support the motor nature of the nerves studied. The LATN showed a significantly higher percentage of 6.5 μm fibers compared with the MATN, but not to the quadriceps nerve: this finding suggests a higher amount of sensory fibers, even if we cannot exclude that these fibers may originate from gamma motor neurons.

Although immunohistochemical staining for ChAT confirmed that all nerves studied are predominantly composed of motor fibers, MATN shows the higher percentage. The quadriceps nerve is commonly considered a predominantly motor nerve and is frequently used in order to assess the motor fiber involvement at hind-limbs (Wrabetz et al., 2000; Kobsar et al., 2003; Grohmann et al., 2004). Even if no systematic study has so far precisely assessed its fiber composition, the percentage of motor fibers has been estimated to be as high as 50% in the mouse (Grohmann et al., 2004). In our hands, the mean percentage of motor fibers was 67% for the quadriceps nerve, non-dissimilar to the LATN (61.2%). The MATN, however, contained a significantly higher percentage of motor fibers (88.8%).

In conclusion, our study yields a systematic description of the branches of the brachial plexus to the pectoral muscles, and might provide reference values for future experimental studies in rats. Moreover, we demonstrated that the MATN and the LATN are predominantly motor nerves and could be used to assess the PNS involvement in diseases predominantly affecting the motor compartment.

### Conflict of interest statement

The authors declare that the research was conducted in the absence of any commercial or financial relationships that could be construed as a potential conflict of interest.
